# Structure and comparison of the motor domain of centromere-associated protein E

**DOI:** 10.1107/S2059798321000176

**Published:** 2021-02-17

**Authors:** Asuka Shibuya, Naohisa Ogo, Jun-ichi Sawada, Akira Asai, Hideshi Yokoyama

**Affiliations:** a Faculty of Pharmaceutical Sciences, Tokyo University of Science, 2641 Yamazaki, Noda, Chiba 278-8510, Japan; b Center for Drug Discovery, Graduate School of Pharmaceutical Sciences, University of Shizuoka, 52-1 Yada, Suruga-ku, Shizuoka 422-8526, Japan

**Keywords:** CENP-E, anticancer drugs, kinesins

## Abstract

Centromere-associated protein E (CENP-E) plays an essential role in mitosis and is a target candidate for anticancer drugs. However, it is difficult to design small-molecule inhibitors of CENP-E kinesin motor ATPase activity owing to a lack of structural information on the CENP-E motor domain in complex with its inhibitors. Here, the CENP-E motor domain was crystallized in the presence of an ATP-competitive inhibitor and the crystal structure was determined at 1.9 Å resolution. In the determined structure, ADP was observed instead of the inhibitor in the nucleotide-binding site, even though no ADP was added during protein preparation. Structural comparison with the structures of previously reported CENP-E and those of other kinesins indicates that the determined structure is nearly identical except for several loop regions. However, the retention of ADP in the nucleotide-binding site of the structure strengthens the biochemical view that the release of ADP is a rate-limiting step in the ATPase cycle of CENP-E. These results will contribute to the development of anticancer drugs targeting CENP-E and to understanding the function of kinesin motor domains.

## Abstract 2

Crystallization and structure determination of the motor domain of centromere-associated protein E in complex with its inhibitor was performed. In the determined structure, endogenous ADP was observed in the nucleotide-binding site instead of the inhibitor.

## 1. Introduction   

Antimitotic anticancer drugs, such as taxanes and vinca alkaloids, have been widely used in the clinical therapy of human malignancies (Wood *et al.*, 2001 ▸; Jordan & Wilson, 2004 ▸). They cause serious side effects such as toxicity in non­dividing cells such as peripheral neurons. On the other hand, antimitotic agents that target mitotic kinesins are expected to be more likely to act on dividing cells but not on nondividing cells, and thus antimitotic agents that inhibit the functions of kinesin motor domains minimize the toxicity to nondividing cells, causing decreased side effects. Therefore, such inhibitors will be promising candidates for the development of cancer drugs (Sakowicz *et al.*, 2004 ▸). To date, a large number of Eg5 inhibitors, such as STLC and PVZB1194, have been reported (Ogo *et al.*, 2007 ▸; Matsuno *et al.*, 2009 ▸). A large amount of structural information on Eg5 in complex with its inhibitors has also been reported (Yokoyama *et al.*, 2015 ▸, 2018 ▸; Myers & Collins, 2016 ▸). However, Eg5 inhibitors have not found clinical use because they target not only cancer cells but also other actively proliferating cells.

A recent new target for mitotic inhibition is centromere-associated protein E (CENP-E), which is a member of the kinesin-7 subfamily. CENP-E plays important roles in proper chromosome segregation during mitosis. The role of CENP-E in chromosome congression has been extensively described in various models (McEwen *et al.*, 2001 ▸; Putkey *et al.*, 2002 ▸; Schaar *et al.*, 1997 ▸; Wood *et al.*, 1997 ▸; Yao *et al.*, 2000 ▸). After entering mitosis, CENP-E locates on the kinetochores during spindle formation, and is required for the proper alignment of mitotic chromosomes at the spindle midzone (Wood *et al.*, 1997 ▸; Schaar *et al.*, 1997 ▸; Kim *et al.*, 2010 ▸). The motor domain of CENP-E plays an important role in transporting peripheral polar chromosomes towards the spindle midzone (Barisic *et al.*, 2014 ▸; Zhang *et al.*, 2017 ▸). CENP-E is composed of three domains: tail, stalk and motor. The motor domain, located at the N-terminus, consists of 339 residues and is approximately 40 kDa in size; it is the active site for ATPase activity. CENP-E uses the N-terminal motor domain to gain driving energy by hydrolyzing adenosine triphosphate (ATP) to adenosine diphosphate (ADP), and moves along microtubules. The release of ADP from CENP-E is significantly slower than from other kinesins and is the rate-limiting step in ATP turnover (Sardar & Gilbert, 2012 ▸).

To date, only the crystal structure of the CENP-E motor domain in complex with MgADP (CENP-E–MgADP 1t5c) has been reported (Garcia-Saez *et al.*, 2004 ▸; PDB entry 1t5c). It is difficult to perform rational drug design by fragment-based drug discovery (FBDD) or structure-based drug design (SBDD) owing to a lack of structural information on CENP-E. Therefore, it is necessary to determine crystal structures of the CENP-E motor domain in complex with its inhibitors.

Here, in order to elucidate the mechanism by which the CENP-E motor domain binds to its inhibitors, we tried to cocrystallize the CENP-E motor domain in complex with the ligand 3-chloro-4-isopropoxyl benzoic acid (CIBA; Qian *et al.*, 2010 ▸), an ATP-competitive inhibitor, and determined the structure at 1.9 Å resolution. Endogenous ADP instead of CIBA was observed in the nucleotide-binding site, even though ATP or ADP had not been added. The determined structure of the CENP-E motor domain was compared with those of other kinesin motors. Based on the characteristic structure of CENP-E, the mechanism by which ADP is retained in CENP-E is discussed.

## 2. Materials and methods   

### 2.1. Preparation of inhibitor and construction of plasmids   

CIBA was synthesized as described by Qian *et al.* (2010 ▸). The cDNA of CENP-E_1–339_ (residues 1–339 of CENP-E) was cloned into pCold III bacterial expression vector to construct pCENP-E_1–339_, similarly to as described by Yamane *et al.* (2019 ▸). The recombinant protein consisted of the CENP-E motor domain (Met1–Ser339) extended with MNHKVH at the N-terminus and GSHHHHHH at the C-terminus.

### 2.2. Protein preparation of CENP-E constructs   

Wild-type CENP-E_1–339_ with extra residues was expressed in *Escherichia coli* BL21 (DE3) CodonPlus RIL cells as a C-terminal His_6_-fusion protein. The *E. coli* BL21 (DE3) CodonPlus RIL cells (Stratagene) were transformed with the plasmid and were grown at 37°C in 2YT medium containing 1.6% Bacto Tryptone (Nacalai), 1.0% yeast extract (Nacalai) and 0.5% NaCl (Wako) in the presence of 0.1 mg ml^−1^ ampicillin (Nacalai) and were induced with 0.4 m*M* isopropyl β-d-1-thiogalactopyranoside (IPTG; Nacalai) at 15°C overnight.

The recombinant protein was purified in three steps involving nickel-affinity, cation-exchange and gel-filtration chromatography. The harvested cells were resuspended in buffer consisting of 50 m*M* Tris–HCl pH 7.5, 0.5 *M* NaCl, 2 m*M* MgCl_2_, 0.2 m*M* EGTA, 5 m*M* β-mercaptoethanol, 25 m*M* imidazole, 10%(*w*/*v*) sucrose and protease-inhibitor cocktail (Roche) and adjusted to pH 7.4, and were disrupted by sonication. The cell lysate was centrifuged and the supernatant was loaded onto 1 ml Ni–NTA agarose resin (Qiagen) equilibrated with buffer consisting of 20 m*M* Tris–HCl, 0.3 *M* NaCl, 2 m*M* MgCl_2_, 5 m*M* β-mercaptoethanol, 20 m*M* imidazole, 10%(*w*/*v*) sucrose and adjusted to pH 7.4. After washing with buffer containing 25 m*M* imidazole, the proteins were eluted with buffer consisting of 500 m*M* imidazole, 50 m*M* piperazine-1,4-bis(2-ethanesulfonic acid) (PIPES)–NaOH, 0.1 *M* NaCl, 2 m*M* MgCl_2_, 5 m*M* β-mercaptoethanol, 10%(*w*/*v*) sucrose and adjusted to pH 6.8. The eluted protein was loaded onto a 1 ml HiTrap SP HP cation-exchange column (GE Healthcare) equilibrated with buffer consisting of 50 m*M* PIPES–NaOH pH 6.8, 2 m*M* MgCl_2_, 1 m*M* EGTA, 1 m*M* tris(2-carboxy­ethyl)phosphine (TCEP), 5%(*w*/*v*) sucrose and adjusted to pH 6.8, and was eluted with a linear gradient of 0–0.4 *M* NaCl. The eluted fractions were further purified by gel-filtration chromatography using a HiLoad 16/600 Superdex 200 prep-grade column equilibrated with buffer consisting of 50 m*M* PIPES–NaOH pH 6.8, 2 m*M* MgCl_2_, 1 m*M* EGTA, 1 m*M* TCEP, 5%(*w*/*v*) sucrose, 0.3 *M* NaCl and adjusted to pH 6.8. The eluted proteins were concentrated with a Vivaspin 20 centrifugal concentrator (Sartorius) with a 10 kDa molecular-mass cutoff. The concentration of CENP-E was determined with a NanoDrop One (Thermo Scientific) using an extinction coefficient of 3.186 × 10^4^ *M* ^−1^ cm^−1^. The purity of the CENP-E protein during the purification procedure was confirmed by SDS–PAGE analysis.

### 2.3. Crystallization   

The purified protein was at 11 mg ml^−1^ in 50 m*M* PIPES–NaOH pH 6.8, 300 m*M* NaCl, 2 m*M* MgCl_2_, 1 m*M* EGTA, 1 m*M* TCEP, 5%(*w*/*v*) sucrose. Crystallization was performed using the hanging-drop vapor-diffusion method at 4°C. After approximately ten days, imperfect crystals appeared. It has been reported that good crystals can be obtained using the microseed matrix seeding method (D’Arcy *et al.*, 2014 ▸). The 11 mg ml^−1^ protein solution was mixed with CIBA in a molar ratio of 1:10 (at least 277 µ*M* CENP-E_1–339_ and 2.77 m*M* CIBA). Crystallization was performed using the sitting-drop vapor-diffusion method at 4°C. Crystallization drops were prepared by mixing 0.9 µl of the CENP-E_1–339_–CIBA solution described above, 0.8 µl reservoir solution and 0.3 µl seed solution. The seed solution was prepared using the reservoir solution consisting of 90 m*M* Tris–HCl pH 7.5, 18%(*w*/*v*) PEG 3350. Hexahedron-shaped crystals appeared with approximate dimensions of 0.1 × 0.2 × 0.05 mm.

### 2.4. X-ray data collection and structure determination   

A crystal was cryoprotected in a solution consisting of 50 m*M* PIPES–NaOH pH 6.8, 2 m*M* MgCl_2_, 1 m*M* TCEP, 1 m*M* EGTA, 18%(*w*/*v*) PEG 3350, 90 m*M* Tris–HCl pH 7.5, 5%(*w*/*v*) sucrose, 2.77 m*M* CIBA, 22%(*w*/*v*) glycerol and flash-cooled at 95 K. X-ray diffraction data were collected on beamline BL17A at the Photon Factory, KEK, Tsukuba, Japan and were processed and scaled with *XDS* (Kabsch, 2010 ▸) and *SCALA* (Evans, 2006 ▸). The structure was determined by the molecular-replacement method using *MOLREP* (Vagin & Teplyakov, 2010 ▸) in the *CCP*4 suite (Winn *et al.*, 2011 ▸). The structure of CENP-E–MgADP 1t5c (Garcia-Saez *et al.*, 2004 ▸; PDB entry 1t5c) was used as an initial model. Structural refinement was performed with *REFMAC*5 (Murshudov *et al.*, 2011 ▸) and *Phenix* (Liebschner *et al.*, 2019 ▸). Manual model fitting was achieved with *Coot* (Emsley *et al.*, 2010 ▸). Data-collection and refinement statistics are summarized in Table 1 ▸. Ramachandran statistics were calculated with *MolProbity* (Chen *et al.*, 2010 ▸). Least-squares fitting between two structures was performed with *PDBeFold* (https://www.ebi.ac.uk/msd-srv/ssm/) using all residues. All molecular figures were produced with *PyMOL* (http://www.pymol.org/).

## 3. Results and discussion   

### 3.1. Structure determination   

**Figure 1:**
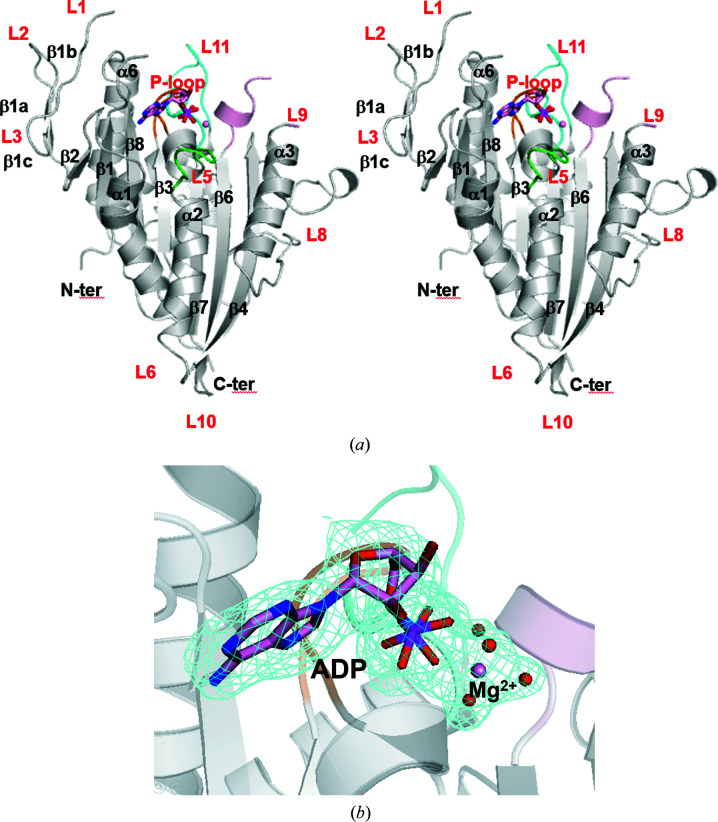
Structure of human CENP-E. (*a*) A front view of CENP-E–MgADP from this study is shown in cartoon representation (stereoview). The P-loop (orange), L5 (green), L9 (light pink), L11 (cyan), ADP and Mg (pink) are shown. (*b*) The *F*_o_ − *F*_c_ OMIT map for ADP, Mg^2+^ and water molecules is depicted. The *F*_o_ − *F*_c_ OMIT map was calculated with the phases from the model without ADP, Mg^2+^ and neighboring water molecules and contoured at 3σ.

We tried to determine the structure of the CENP-E motor domain in complex with its inhibitor CIBA. The structure was determined at 1.9 Å resolution, which was higher than that of the previously reported structure CENP-E–MgADP 1t5c (Garcia-Saez *et al.*, 2004 ▸; PDB entry 1t5c; 2.5 Å resolution; Fig. 1 ▸ *a*). Unfortunately, electron density for ADP instead of CIBA was observed in the nucleotide-binding site, although ADP had not been added during protein preparation (Fig. 1 ▸ *b*). The determined structure was of the CENP-E motor domain in complex with MgADP. There are two molecules (chains *A* and *B*) in the asymmetric unit. The structure from this study is almost identical to the previously reported structure CENP-E–MgADP 1t5c (Garcia-Saez *et al.*, 2004 ▸; PDB entry 1t5c), with a root-mean-square deviation (r.m.s.d.) for corresponding C^α^ atoms of 0.71 Å. Gel-filtration analysis has suggested that the CENP-E motor domain is monomeric (Garcia-Saez *et al.*, 2004 ▸). Hereafter, molecule *A* is used to discuss the structure of the CENP-E motor domain.

Molecule *A* includes residues Glu4–Asn17, Ala27–Asn159, Asn161–Tyr191, Asn197–Lys216, Gly224–Ala243 and Leu252–Ser339 and MgADP. Molecule *B* comprises residues Glu4–Ser18, Ala27–Tyr191, Gln198–Lys216, Ser225–Ala243, Leu252–Gln276 and Phe280–Ser339 and MgADP. The C^α^ atoms of 301 residues in the two monomers were superposed by a least-squares fit using *PDBeFold* and their final r.m.s.d. was 0.28 Å. The average *B* factor of the protein was relatively high compared with the Wilson *B* factor (Table 1 ▸). This may be because the structure contains a large number of disordered and missing residues.

### 3.2. Overall structure   

Fig. 1 ▸(*a*) shows a front view of the CENP-E–MgADP structure reported in this study. It has a mixed eight-stranded β-sheet core with flanking solvent-exposed α-helices and a small three-stranded antiparallel β-sheet in the N-terminal region (β1a, β1b and β1c). The long linker region of β9 and β10 (Fig. 1 ▸ *a*) has the same docked conformation as described in the previously reported structure of the CENP-E motor domain (Garcia-Saez *et al.*, 2004 ▸).

### 3.3. Structural comparison with known structures   

**Figure 2:**
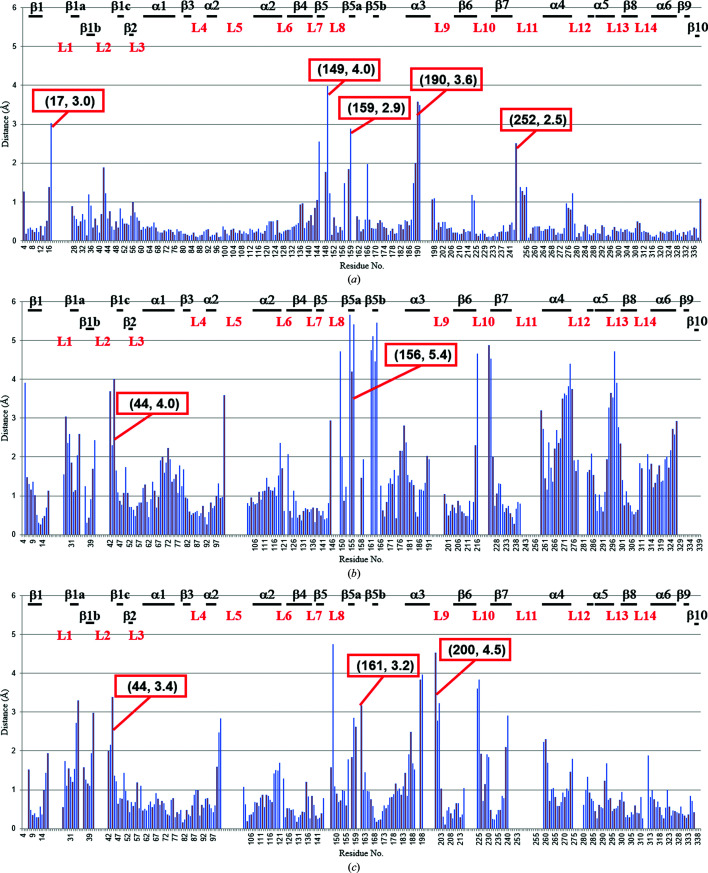
Plots of C^α^-atom distances relative to other structures. Using *PDBeFold*, chain *A* of the structure of CENP-E–MgADP from this study was individually superposed onto chain *A* of three types of kinesins. The corresponding C^α^-atom distances between CENP-E–MgADP from this study and superposed CENP-E–MgADP (PDB entry 1t5c) (*a*), Eg5–MgADP (PDB entry 1ii6) (*b*) and Eg5–MgAMPPNP (PDB entry 3hqd) (*c*) are shown. The corresponding secondary-structure and loop elements are labeled. The residue number and r.m.s.d. value are shown in parentheses.

The structure of chain *A* of CENP-E–MgADP reported in this study was compared with the previously determined structures of CENP-E–MgADP (Garcia-Saez *et al.*, 2004 ▸; PDB entry 1t5c), the motor domain of Eg5 in complex with MgADP (Eg5–MgADP; Turner *et al.*, 2001 ▸; PDB entry 1ii6) and Eg5–AMPPNP (Parke *et al.*, 2010 ▸; PDB entry 3hqd) (Fig. 2 ▸). The CENP-E–MgADP structure determined at 1.9 Å resolution in this study will provide significantly more structural information than the previously determined CENP-E–MgADP 1t5c structure at 2.5 Å resolution. Several similar and differing features of the two structures are described. The structure in this study differed slightly from that of CENP-E–MgADP 1t5c. Loop L1 has been claimed to be unique to CENP-E (Garcia-Saez *et al.*, 2004 ▸). The α0 helix is a conserved structure in kinesins such as Eg5. The structure in this study is nearly identical to CENP-E–MgADP 1t5c but these residues are disordered (Figs. 2 ▸ and 4*a*). The region containing α0 and L1 of CENP-E seems to be flexible, which indicates that this structure is unique to the CENP-E motor domain.

The structure at the beginning of L2 (residues 41–45 of chain *A*) differs from those in Eg5–MgADP and Eg5–MgAMPPNP but is nearly identical to that in CENP-E–MgADP 1t5c (Figs. 2 ▸ and 4*a*). L2 in CENP-E–MgADP is smaller than those in Eg5–MgADP and Eg5–AMPPNP. The loop itself has a double conformation. Thus, each of the residues Asp34–Asn36 of L2 in CENP-E–MgADP from this study has a double conformation. His54 of L3 in CENP–MgADP from this study has a double conformation.

**Figure 3:**
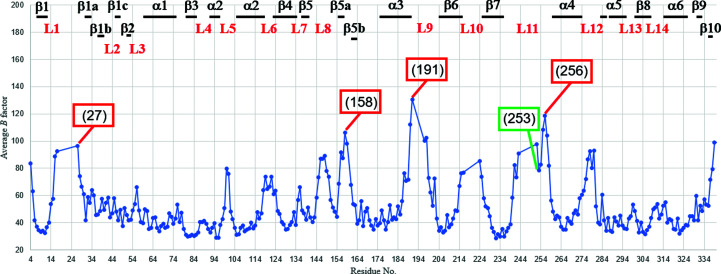
Plots of average *B* factors of each residue of chain *A* of CENP-E–MgADP from this study. Residue numbers are given in parentheses.

The orientation of the side chain of His111 in the middle of α2 is the same as that in CENP-E–MgADP 1t5c but differs from those in Eg5–MgADP and Eg5–AMPPNP. Therefore, it is unique to CENP-E. The *B* factors of the main chain and side chain of His111 are below 40 Å^2^ (Fig. 3 ▸). The beginning of α2 is in almost the same position as in other kinesins although it is close to the nucleotide-binding site. The orientation of the α2 helix is similar to that in other kinesins such as Eg5 (Figs. 2 ▸ *b*, 2 ▸ *c* and 4 ▸ *b*). The end of α2 of CENP-E–MgADP from this study is away from the eight-stranded β-sheet core. Although the number of residues in α2 is the same, the large r.m.s.d. value indicates that the α-helix is slightly shrunk compared with those of Eg5–MgADP and Eg5–MgAMPPNP. The r.m.s.d. for CENP-E–MgADP 1t5c is up to 0.6 Å, indicating that the structure of α2 is unique to CENP-E (Fig. 2 ▸ *a*).

**Figure 4:**
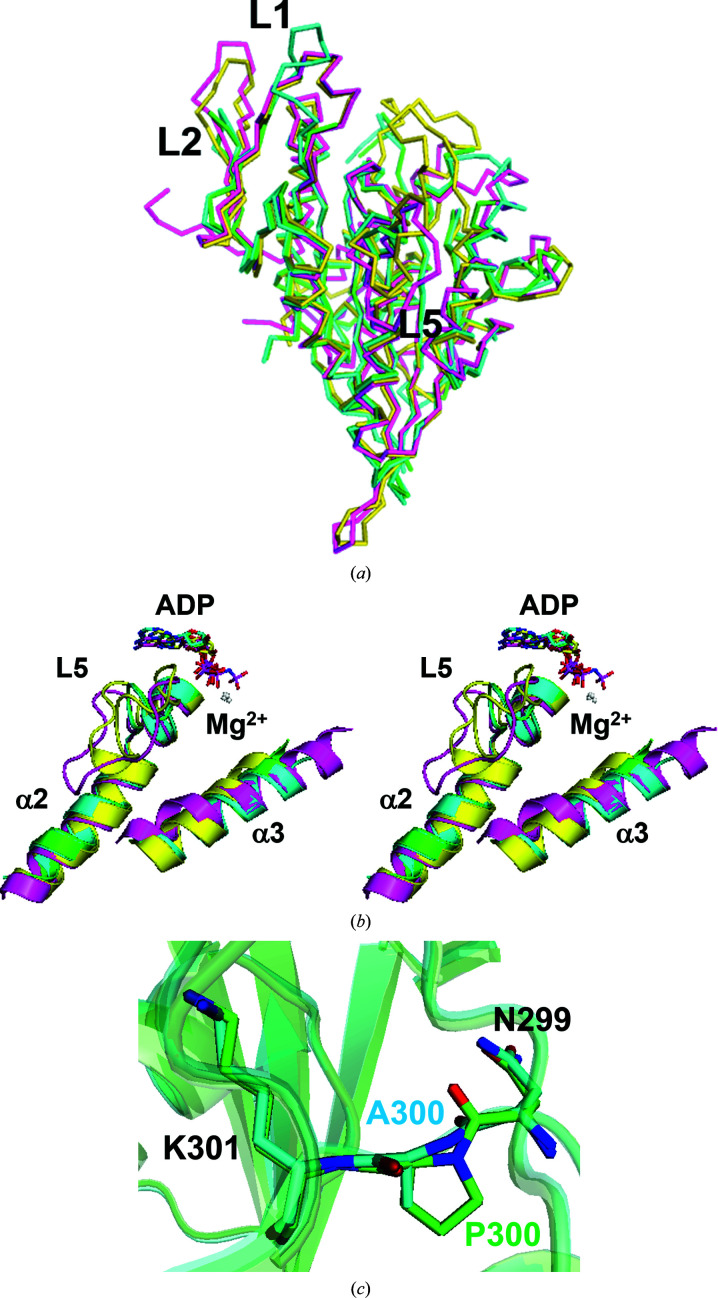
Structural comparison with other structures. The structures of CENP-E–MgADP from this study (green), CENP-E–MgADP 1t5c (cyan), Eg5–MgADP (PDB entry 1ii6; magenta) and Eg5–MgAMPPNP (PDB entry 3hqd; yellow) and Mg^2+^ (white) are shown. (*a*) Ribbon representations of the structure of the CENP-E–MgADP from this study superposed with the previously reported structures of CENP-E–MgADP 1t5c, Eg5–MgADP (PDB entry 1ii6) and Eg5–MgAMPPNP (PDB entry 3hqd). The view is the same as in Fig. 1 ▸(*a*). (*b*) CENP-E has a unique structural orientation of L5 and helix α3 compared with ADP-bound and ATP-bound forms of Eg5 (stereoview). (*c*) Stick representations of superposed residues 299–301 of CENP-­E–MgADP from this study and CENP-E–MgADP 1t5c.

The structure of L5, which is located in the middle of α2 in kinesin motor domains and is involved in the binding of inhibitor by Eg5, is nearly identical to that of CENP-E–MgADP 1t5c and is smaller than those of Eg5–MgADP and Eg5–AMPPNP, as described in the previous report (Garcia-Saez *et al.*, 2004 ▸; Fig. 4 ▸ *b*). L5 of Eg5 regulates both nucleotide and microtubule binding through a set of reversible inter­actions with α3 (Muretta *et al.*, 2013 ▸). His102 of CENP-E–MgADP from this study also has a double conformation.

The loop between the end of β5 and the beginning of β5b (residues 144–161), which is not conserved in kinesins, differs from those in CENP-E–MgADP 1t5c, Eg5–ADP and Eg5–MgAMPPNP (Fig. 2 ▸). Most of these residues have average *B* factors of over 55 Å^2^ (Fig. 3 ▸). The structure of the region between β5a and β5b (residues 156–161) differs from those in CENP-E–MgADP 1t5c, Eg5–MgADP and Eg5–MgAMPPNP (Fig. 2 ▸).

Helix α3 is relatively similar in the structures of CENP-E–MgADP 1t5c and Eg5–MgAMPPNP (Figs. 2 ▸ *a* and 2 ▸ *c*). The orientation of α3 is similar to that in CENP-E–MgADP 1t5c, but the positions of both ends of α3 are closer to the β-sheet core and are intermediate between the previously reported structures of Eg5–MgADP and Eg5–MgAMPPNP. Helix α3 of CENP-E–MgADP from this study has a larger number of residues than that of Eg5–MgAMPPNP.

L9 and L11, which correspond to switch I and II, respectively, of CENP-E–MgADP from this study, show high *B* factors (Fig. 3 ▸) and were not modeled. Owing to high flexibility, the structure differs from those of CENP-E–MgADP 1t5c, Eg5–MgADP and Eg5–MgAMPPNP. The r.m.s.d. values for the region 199–203 between the structure of CENP-E–MgADP from this study and Eg5–MgAMPPNP are high (Fig. 2 ▸ *c*), suggesting that this region is involved in the nucleotide-binding site. The structure of the end of L11 was also unique to other kinesin structures. The average *B* factors of residues 252–258 (L11) were high, so these regions were flexible (Fig. 3 ▸). However, the structure of residues 252–254, which interacted with the symmetry-related molecule, was located in a slightly different position compared with CENP-E–MgADP 1t5c. This is a possible reason why the *B* factor of residue 253 is relatively low (Fig. 3 ▸).

The structure of L13 between α5 and β8 in this study is nearly identical to that in CENP-E–MgADP 1t5c, but not to that in Eg5–MgADP (Fig. 2 ▸), indicating that the main chain of residues 287–301 in this long region was unique to the CENP-E motor domain.

CENP-E–MgADP 1t5c contained a P300A mutation, whereas the CENP-E motor domain in this study was expressed as the wild type. Pro300 is unique to CENP-E in kinesins. The main chain of Pro300 and the peptide bond between residues 299 and 300 of CENP-E–MgADP from this study is located in almost the same position as that in CENP-E–MgADP 1t5c (Fig. 4 ▸ *c*).

### 3.4. Nucleotide-binding site   

**Figure 5:**
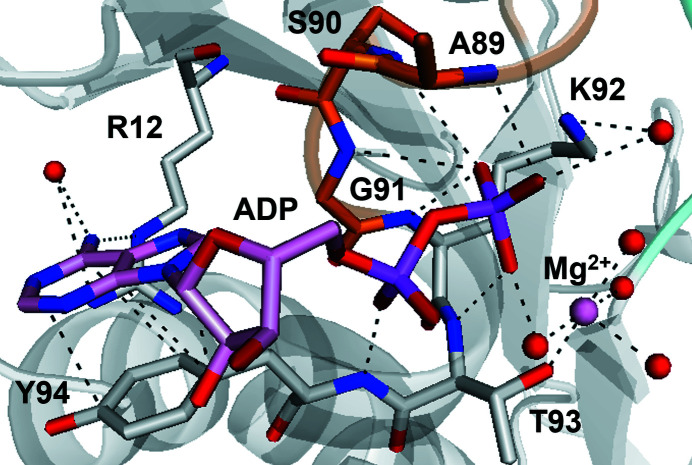
Nucleotide-binding site. Tyr94 interacts with ADP more strongly than in other kinesins owing to the greater abundance of electrons in the aromatic ring. Dashed lines indicate interactions of shorter than 4.0 Å. Red spheres show water molecules.

MgADP and four water molecules are located in the nucleotide-binding pocket (Figs. 1 ▸ *b*, 4 ▸ *b* and 5 ▸). The Mg^2+^ ion interacts with a β-phosphate oxygen moiety, four water molecules and Thr93 O^γ1^ at the end of the P-loop (Fig. 5 ▸). The adenosine ring moiety of ADP makes van der Waals inter­actions with the side chain of Tyr94 at the beginning of α2 of CENP-E (Fig. 5 ▸) or Phe in Eg5. At the nucleotide-binding site of CENP-E, hydrophobic interactions such as π–π stacking between ADP and Tyr94 are stronger than those in other kinesins. This is expected to be one potential reason why CENP-E tends to retain ADP for a long time. α2 and L5 located near ADP are shorter than in other kinesins, which is unique to CENP-E (Figs. 4 ▸ *a* and 4 ▸ *b*).

Owing to the larger number of residues in loop L1, which includes residues 18–26, compared with other kinesins, we think that the conserved residues 16–27 are disordered and are then located where they are able to interact with ADP. Further studies will be needed to support the possibility.

The CENP-E motor domain used in this study exhibited ATPase activity in the presence and absence of microtubules in an ATPase assay performed at 25°C. For crystal structure analysis, the CENP-E motor domain was prepared at 4°C without adding ATP/ADP; CIBA was then added after several days of purification. However, the structure of CENP-E–MgADP from this study contained ADP but not CIBA (Figs. 1 ▸, 4 ▸ *b* and 5 ▸ *a*). The ADP was derived from the bacteria used to express the CENP-E motor domain, and CENP-E is thought to have retained ADP throughout the purification procedure. This suggests that ADP release may be a control point in the role of CENP-E in chromosome congression during mitosis.

## 4. Conclusion   

The structure was determined by crystallizing the CENP-E motor domain with its inhibitor CIBA. However, electron density for ADP instead of CIBA was observed in the nucleotide-binding site of CENP-E, even though ADP was not added during protein preparation. The determined structure at 1.9 Å resolution shows the CENP-E motor domain in complex with MgADP, and also gives much more structural information than the previously reported structure at 2.5 Å resolution. The results of this study support the biochemical view that the release of ADP from CENP-E is a rate-limiting step in the ATPase cycle (Sardar & Gilbert, 2012 ▸). In order to elucidate the structure of the CENP-E motor domain bound to its ATP-competitive inhibitor, it will be necessary to perform an additional experimental procedure to dissociate ADP from CENP-E before forming a CENP-E–inhibitor complex. Further studies will be needed to clarify the mechanism by which ADP dissociates from CENP-E.

This structure will contribute to understanding and clarifying the function of the kinesin CENP-E. Studies of CENP-E will also lead to the development of anticancer drugs and will be of considerable interest for future antimitotic therapies.

## Supplementary Material

PDB reference: CENP-E motor domain, 6m4i

## Figures and Tables

**Table 1:** Data-collection and refinement statistics Values in parentheses are for the outer shell.

Data collection	
Resolution range (Å)	20.00–1.90
Wavelength (Å)	0.9800
Space group	*P*2_1_
*a*, *b*, *c* (Å)	96.8, 82.8, 49.4
α, β, γ (°)	90, 101, 90
Total No. of reflections	414345 (61149)
No. of unique reflections	60258 (8718)
Multiplicity	6.88
*R*_merge_(*I*)†	0.048 (0.830)
Completeness (%)	99.8 (99.8)
Average 〈*I*/σ(*I*)〉	18.2 (2.6)
*R*_meas_	0.052 (0.897)
Wilson *B* factor (Å^2^)	38.1
Refinement	
No. of reflections	54214
*R*/*R*_free_ ‡	0.217/0.255
No. of non-H atoms	
Protein	4924
Ligand	56
Water	140
Average *B* factor (Å^2^)	
Protein	60.6
Ligand	50.8
Water	50.6
R.m.s. deviations	
Bonds (Å)	0.009
Angles (°)	1.559
Ramachandran plot	
Most favored (%)	97.6
Allowed (%)	2.4
Outliers (%)	0
PDB code	6m4i

† *R*_merge_(*I*) = 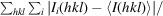 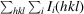, where *I_i_*(*hkl*) is the intensity of an individual reflection and 〈*I*(*hkl*)〉 is the mean intensity of that reflection.

‡ *R* = 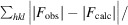 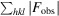, where |*F*_obs_| and |*F*_calc_| are the observed and calculated structure-factor amplitudes, respectively. *R*_free_ is calculated for 10% of the reflections that were randomly excluded from refinement.

## References

[bb2] Barisic, M., Aguiar, P., Geley, S. & Maiato, H. (2014). *Nat. Cell Biol.* **16**, 1249–1256. 10.1038/ncb3060 25383660

[bb3] Chen, V. B., Arendall, W. B., Headd, J. J., Keedy, D. A., Immormino, R. M., Kapral, G. J., Murray, L. W., Richardson, J. S. & Richardson, D. C. (2010). *Acta Cryst.* D**66**, 12–21. 10.1107/S0907444909042073 PMC2803126 20057044

[bb4] D’Arcy, A., Bergfors, T., Cowan-Jacob, S. W. & Marsh, M. (2014). *Acta Cryst.* F**70**, 1117–1126. 10.1107/S2053230X14015507 PMC4157405 25195878

[bb5] Emsley, P., Lohkamp, B., Scott, W. G. & Cowtan, K. (2010). *Acta Cryst.* D**66**, 486–501. 10.1107/S0907444910007493 PMC2852313 20383002

[bb6] Evans, P. (2006). *Acta Cryst.* D**62**, 72–82. 10.1107/S0907444905036693 16369096

[bb7] Garcia-Saez, I., Yen, T., Wade, R. H. & Kozielski, F. (2004). *J. Mol. Biol.* **340**, 1107–1116. 10.1016/j.jmb.2004.05.053 15236970

[bb8] Jordan, M. A. & Wilson, L. (2004). *Nat. Rev. Cancer*, **4**, 253–265. 10.1038/nrc1317 15057285

[bb9] Kabsch, W. (2010). *Acta Cryst.* D**66**, 125–132. 10.1107/S0907444909047337 PMC2815665 20124692

[bb10] Kim, Y., Holland, A. J., Lan, W. & Cleveland, D. W. (2010). *Cell*, **142**, 444–455. 10.1016/j.cell.2010.06.039 PMC2921712 20691903

[bb1] Liebschner, D., Afonine, P. V., Baker, M. L., Bunkóczi, G., Chen, V. B., Croll, T. I., Hintze, B., Hung, L.-W., Jain, S., McCoy, A. J., Moriarty, N. W., Oeffner, R. D., Poon, B. K., Prisant, M. G., Read, R. J., Richardson, J. S., Richardson, D. C., Sammito, M. D., Sobolev, O. V., Stockwell, D. H., Terwilliger, T. C., Urzhumtsev, A. G., Videau, L. L., Williams, C. J. & Adams, P. D. (2019). *Acta Cryst.* D**75**, 861–877. 10.1107/S2059798319011471 PMC6778852 31588918

[bb11] Matsuno, K., Sawada, J., Sugimoto, M., Ogo, N. & Asai, A. (2009). *Bioorg. Med. Chem. Lett.* **19**, 1058–1061. 10.1016/j.bmcl.2009.01.018 19167222

[bb12] McEwen, B. F., Chan, G. K. T., Zubrowski, B., Savoian, M. S., Sauer, M. T. & Yen, T. J. (2001). *Mol. Biol. Cell*, **12**, 2776–2789. 10.1091/mbc.12.9.2776 PMC59712 11553716

[bb13] Muretta, J. M., Behnke-Parks, W. M., Major, J., Petersen, K. J., Goulet, A., Moores, C. A., Thomas, D. D. & Rosenfeld, S. S. (2013). *J. Biol. Chem.* **288**, 34839–34849. 10.1074/jbc.M113.518845 PMC3843096 24145034

[bb14] Murshudov, G. N., Skubák, P., Lebedev, A. A., Pannu, N. S., Steiner, R. A., Nicholls, R. A., Winn, M. D., Long, F. & Vagin, A. A. (2011). *Acta Cryst.* D**67**, 355–367. 10.1107/S0907444911001314 PMC3069751 21460454

[bb15] Myers, M. S. & Collins, I. (2016). *Future Med. Chem.* **8**, 463–489. 10.4155/fmc.16.5 PMC4896392 26976726

[bb16] Ogo, N., Oishi, S., Matsuno, K., Sawada, J., Fujii, N. & Asai, A. (2007). *Bioorg. Med. Chem. Lett.* **17**, 3921–3924. 10.1016/j.bmcl.2007.04.101 17524640

[bb17] Parke, C. L., Wojcik, E. J., Kim, S. & Worthylake, D. K. (2010). *J. Biol. Chem.* **285**, 5859–5867. 10.1074/jbc.M109.071233 PMC2820811 20018897

[bb18] Putkey, F. R., Cramer, T., Morphew, M. K., Silk, A. D., Johnson, R. S., McIntosh, J. R. & Cleveland, D. W. (2002). *Dev. Cell*, **3**, 351–365. 10.1016/s1534-5807(02)00255-1 12361599

[bb19] Qian, X., McDonald, A., Zhou, H. J., Adams, N. D., Parrish, C. A., Duffy, K. J., Fitch, D. M., Tedesco, R., Ashcraft, L. W., Yao, B., Jiang, H., Huang, J. K., Marin, M. V., Aroyan, C. E., Wang, J., Ahmed, S., Burgess, J. L., Chaudhari, A. M., Donatelli, C. A., Darcy, M. G., Ridgers, L. H., Newlander, K. A., Schmidt, S. J., Chai, D., Colón, M., Zimmerman, M. N., Lad, L., Sakowicz, R., Schauer, S., Belmont, L., Baliga, R., Pierce, D. W., Finer, J. T., Wang, Z., Morgan, B. P., Morgans, D. J., Auger, K. R., Sung, C. M., Carson, J. D., Luo, L., Hugger, E. D., Copeland, R. A., Sutton, D., Elliott, J. D., Jackson, J. R., Wood, K. W., Dhanak, D., Bergnes, G. & Knight, S. D. (2010). *ACS Med. Chem. Lett.* **1**, 30–34.

[bb20] Sakowicz, R., Finer, J. T., Beraud, C., Crompton, A., Lewis, E., Fritsch, A., Lee, Y., Mak, J., Moody, R., Turincio, R., Chabala, J. C., Gonzales, P., Roth, S., Weitman, S. & Wood, K. W. (2004). *Cancer Res.* **64**, 3276–3280. 10.1158/0008-5472.can-03-3839 15126370

[bb21] Sardar, H. S. & Gilbert, S. P. (2012). *J. Biol. Chem.* **287**, 24894–24904. 10.1074/jbc.M112.376830 PMC3408146 22637578

[bb22] Schaar, B. T., Chan, G. K., Maddox, P., Salmon, E. D. & Yen, T. J. (1997). *J. Cell Biol.* **139**, 1373–1382. 10.1083/jcb.139.6.1373 PMC2132614 9396744

[bb23] Turner, J., Anderson, R., Guo, J., Beraud, C., Fletterick, R. & Sakowicz, R. (2001). *J. Biol. Chem.* **276**, 25496–25502. 10.1074/jbc.M100395200 11328809

[bb24] Vagin, A. & Teplyakov, A. (2010). *Acta Cryst.* D**66**, 22–25. 10.1107/S0907444909042589 20057045

[bb25] Winn, M. D., Ballard, C. C., Cowtan, K. D., Dodson, E. J., Emsley, P., Evans, P. R., Keegan, R. M., Krissinel, E. B., Leslie, A. G. W., McCoy, A., McNicholas, S. J., Murshudov, G. N., Pannu, N. S., Potterton, E. A., Powell, H. R., Read, R. J., Vagin, A. & Wilson, K. S. (2011). *Acta Cryst.* D**67**, 235–242. 10.1107/S0907444910045749 PMC3069738 21460441

[bb26] Wood, K. W., Cornwell, W. D. & Jackson, J. R. (2001). *Curr. Opin. Pharmacol.* **1**, 370–377. 10.1016/s1471-4892(01)00064-9 11710735

[bb27] Wood, K. W., Sakowicz, R., Goldstein, L. S. B. & Cleveland, D. W. (1997). *Cell*, **91**, 357–366. 10.1016/s0092-8674(00)80419-5 9363944

[bb28] Yamane, M., Sawada, J., Ogo, N., Ohba, M., Ando, T. & Asai, A. (2019). *Biochem. Biophys. Res. Commun.* **519**, 505–511. 10.1016/j.bbrc.2019.09.028 31530389

[bb29] Yao, X., Abrieu, A., Zheng, Y., Sullivan, K. F. & Cleveland, D. W. (2000). *Nat. Cell Biol.* **2**, 484–491. 10.1038/35019518 10934468

[bb30] Yokoyama, H., Sawada, J., Katoh, S., Matsuno, K., Ogo, N., Ishikawa, Y., Hashimoto, H., Fujii, S. & Asai, A. (2015). *ACS Chem. Biol.* **10**, 1128–1136. 10.1021/cb500939x 25622007

[bb31] Yokoyama, H., Sawada, J. I., Sato, K., Ogo, N., Kamei, N., Ishikawa, Y., Hara, K., Asai, A. & Hashimoto, H. (2018). *ACS Omega*, **3**, 12284–12294. 10.1021/acsomega.8b00778 PMC6644766 31459302

[bb32] Zhang, H., Aonbangkhen, C., Tarasovetc, E. V., Ballister, E. R., Chenoweth, D. M. & Lampson, M. A. (2017). *Nat. Chem. Biol.* **13**, 1096–1101. 10.1038/nchembio.2456 PMC5605432 28805800

